# Application of Geostatistical Analysis and Random Forest for Source Analysis and Human Health Risk Assessment of Potentially Toxic Elements (PTEs) in Arable Land Soil

**DOI:** 10.3390/ijerph17249296

**Published:** 2020-12-12

**Authors:** Liang Xiao, Yong Zhou, He Huang, Yu-Jie Liu, Ke Li, Meng-Yao Li, Yang Tian, Fei Wu

**Affiliations:** Faculty of Urban and Environmental Sciences, Central China Normal University, Wuhan 430079, China; xiaoliang@mails.ccnu.edu.cn (L.X.); hhuang@mails.ccnu.edu.cn (H.H.); liuyj@mails.ccnu.edu.cn (Y.-J.L.); kli@mails.ccnu.edu.cn (K.L.); limengyao@mails.ccnu.edu.cn (M.-Y.L.); tianyang@mails.ccnu.edu.cn (Y.T.); wfei9527@mails.ccnu.edu.cn (F.W.)

**Keywords:** human health risk assessment, GIS, random forest, arable land, source analysis, China

## Abstract

Arable land soil is one of the most precious natural resources of Earth, it provides the fundamental material and numerous resources essential for the development of human society. To determine the pollution of potential toxic factors in the surface soil of cultivated land and its risks to human health, concentrations of five different potentially toxic elements (PTEs) were detected in 1109 soil samples collected in Xiangzhou, China, in 2019. In this study, health risk assessment was used to judge the degree of pollution in the study area, the result of Geographic Information System (GIS) was as used to research the spatial distribution characteristics of PTEs, and random forest (RF) was used to assess the natural and man-made influencing factors. We investigated the sources of PTEs through quantifying the indicators, which gave further insights. The main results are: (1) In arable land soil, the average content of PTEs is 0.14 mg/kg cadmium (Cd), 0.05 mg/kg mercury (Hg), 12.89 mg/kg arsenic (As), 29.23 mg/kg lead (Pb), and 78.58 mg/kg chromium (Cr), respectively. The content of As and Pb outpaced the background value of Hubei soil. (2) The human health risk assessment in Xiangzhou indicates that the most important exposure pathway is soil ingestion, occupied about 99% to health risks of PTEs; non-carcinogenic risk from exposure to As, Pb and Cr in soil was higher than the limit (overall potential risk index, HI > 1) for both children and adults. Moreover, carcinogenic risk postured by Cd, Cr, and As was higher than the limit (10^−4^) through soil exposure for both children and adults, indicating that Cd, As, Pb and Cr in soil have significant effect on people’s health through exposure. (3) We found that the increased PTEs in the arable land soil mainly originated from potential water sources, air and soil pollution sources, breeding farms, and mining areas.

## 1. Introduction

Arable land soil is fundamental to the survival of animals and plants, representing an important resource and basic element for human survival and development as well as being an important part of the terrestrial ecosystem [1]. Cd, Hg, As, Pb, and Cr are five common potentially toxic elements (PTEs) in arable land [2]. However, when PTEs accumulate in the arable land soil in considerable amounts, leading to a decrease in the quality of the crops and inhibit the life activities of organisms, and pass through the food chain to enter animals and humans, and enter through exposure pathways such as skin contact and inhalation, posing a huge menace to food safety and human health [3].

Human health risk assessment is an important part of environmental impact assessment. Risk assessment combines data from exposure assessment models, toxicology, and epidemiological research to assess the risks and harms which pollutants pose to human health [4]. The study found that exposure to PETs for a long time may lead to health risk to animals and humans [5]. For instance, acute and chronic as exposure would cause an imbalance in the cardiovascular system and other organs, which may finally lead to cancer [6].

As soil contamination by PTEs has been increasing worldwide, source analysis of PTEs has become the focus of attention in recent years. Arao et al. used cluster study to survey the origins of PTEs in soils used for agriculture and tourism in the Zlatibor mountainous area, the study show that Cd was mainly from human sources, and other PTEs were mainly from soil sources [7]. Loska et al. conducted analyses of PTEs in the Sinu River system: the man-made sources of PTEs in the cultivated field in the basin were studied using cluster analysis to determine distinct geographic chemical sets, clustering specimens with similar levels of PTEs pollution into two categories and using principal component analysis to identify factors, such as fertilizers and pesticides, that are related to agricultural production. The migration of pollution was linked with water flow from the upstream metal mine [8].

GIS, random forest, and multivariate statistical analysis can be used to evaluate the spatial allocation of PTEs and determine the source [9]. To make superior use of these tools, investigators are increasingly applying them in combination.

Random forest can be regarded as a classifier containing multiple conditional inference trees. This is a data-mining method based on statistical theory that was developed by Breiman [10]. Random forest was proposed as a fusion algorithm based on decision tree classifiers [11]. The algorithm applies the bootstrap resampling modus to abstract diverse samples from the original samples, establishes a decision tree for each bootstrap sample, and then uses the average of all decision tree predictions as the final prediction result [12]. Random forest improves the prediction accuracy without significantly increasing the number of calculations. This can be used to estimate the importance coefficient of each environmental variable as a means of further identifying the main factors affecting the content of PTEs.

The aim of this research is to assess the concentrations of multiple PTEs in the cultivated soil of Xiangzhou, China, assess the risks to inhabitants’ health resulted by PTEs pollution, and to analyze the source.

## 2. Materials and Methods

### 2.1. Study Location

The research region is Xiangzhou, situated in the northwestern region of Hubei Province. Its geographic location is between 111°44′ and 112°23′ east longitude and 31°46′ and 32°28′ north latitude. The landforms of Xiangzhou are mainly low mountains and hills. The terrain is low in the middle, high in the northwest and southeast, low in the northeast and southwest, and the highest location in the district is 452 m above sea level. The climate of Xiangzhou is a subtropical humid monsoon climate, with simultaneous rain and heat, moderate precipitation, hot and rainy summers, and cold and dry winters. The annual average precipitation is approximately 880 mm; the average temperature during the year is around 15 °C. The main types of land according to use are agricultural land, construction land, and unused land (mainly including waste grassland, saline–alkali land, marshland, sandy land, bare land, bare rock, etc.). The agricultural land is 202,807.25 hectares, accounting for 82.22% of the total land area. Xiangzhou is also a significant grain-producing district in Hubei Province. The high-standard farmland is 70 million hectares, accounting for 34.51% of the whole agricultural land. The soil has a higher overall clay content and moderate pH.

### 2.2. Sampling and Pretreatment

**Figure 1:**
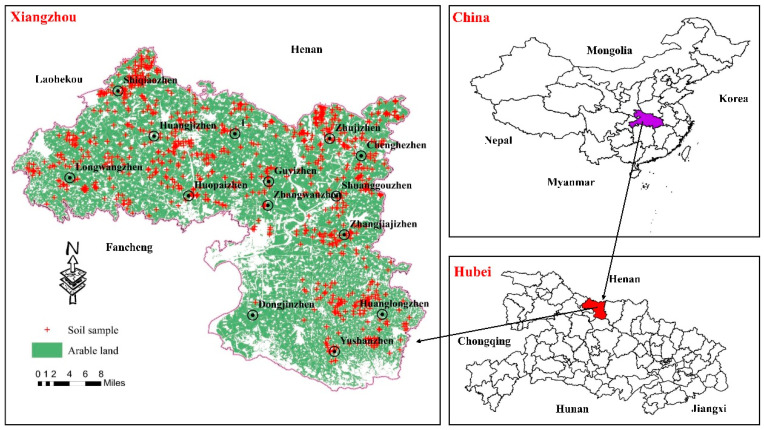
Soil sampling locations in Xiangzhou.

Taking Xiangzhou as the research area and selecting cultivated land as the research object, in the spring dry season of 2019, a total of 1109 soil profiles (numbered XZ-1 to XZ-1109) were collected, as given in Figure 1. The geographic locations of the sampling points were recorded as global positioning system (GPS) coordinates. All soil specimens were packaged in polyethylene sacks, labeled, and the number, location, sampling time, and other information for the sampling points were indicated, and samples were sent to the laboratory for refrigeration. After the samples were air-dried for 30 days in a dry environment, stone and other impurities were sifted out, and the samples were then processed in an oven at 60 °C for 3 h, cooled and stored in a clean, high-density polyethylene (HDPE) bottle for the next analyses.

### 2.3. Digestion of Soil Samples

The specimens were then grinded and passed through a 74 μm riddle. The pH of the soil was measured with a 0.01 mol/L CaCl_2_ solution (soil-to-liquid ratio of 1:5 mixed) for 30 min, then allowed to stand for 1 h and measured with a pH meter. The pretreated sample (0.1 g) was precisely weighed into a polytetrafluoroethylene container, to which 3 mL of HNO_3_, 1 mL of HCl, and 1 mL of HF were added, and then sealed using the microwave chemistry system produced by CEM in the United States. The reaction system, namely a MARS5 microwave digester (Shanghai, China), was used to perform digestion and dissolution. The microwave digestion parameters were power 1600 W, 120 °C for 2 min; 150 °C for 10 min; and 180 °C for 20 min. Following the completion of digestion, specimens were treated at 150 °C to drive away the residual acid until only a small amount was left, and the sample was transferred to a 10 mL measuring flask, in which the digestion tank was washed with a 1% (volume fraction) nitric acid solution, combined with the measuring flask, diluted to the mark, and mixed well for use.

### 2.4. Analytical Procedure

The detection method refers to the standards of “General Principles of Inductively Coupled Plasma Mass Spectrometry Analysis” (DZ/T 0223-2001) and “Determination of Total Mercury, Total Arsenic and Total Lead in Soil” (GB/T 22105-2008). The elements Pb and Cd were analyzed using inductively coupled plasma mass spectrometry (ICP–MS), with a NEXION 350X inductively coupled plasma mass spectrometer (Shanghai, China). Two PTEs, Hg and As, were analyzed by atomic fluorescence spectroscopy (AFS) using the AFS-9700 automatic syringe pump atomic fluorescence spectrometer (Shandong, China). The test was set to 2% parallel samples, the sample contained the reference material sample GBW07402 (GSS-2), the recovery rate of PTEs in the monitoring sample was 90% ± 10%, and the relative deviation of the parallel sample and the recovery rate of the reference material met the quality control requirements.

In order to ensure quality control and assurance, standard operating procedures, using duplicates, reagent blank, spiked samples recovery, several certified reference materials, and standard reference soils (GBW07419; Center for Certified Reference Materials, China) were used. The instrument detection limit (mg/kg) of Cd, Hg, As, Pb, and Cr is: 0.001, 0.001, 0.01, 0.1, and 0.04, respectively. As the existing grinding technology cannot completely crush the soil, the results reflect element contents in the fine fraction of soil. Chemicals were analytical grade (MERCK, Merck Millipore, Darmstadt, Germany).

### 2.5. Health Risk Assessment Methods and Indicators

The National Academy of Sciences (NAS) first proposed a four-step method based on hazard identification, dose–effect evaluation, exposure assessment, and hazard characterization to evaluate the risks of environmental pollutants to human health [13]. The US Environmental Protection Agency (USEPA) subsequently produced a more detailed explanation of the health risk assessment. The health risk assessment of soil pollution with PTEs refers to the assessment of both non-carcinogenic and carcinogenic risks through three routes of exposure, including food intake, skin-surface contact, and breathing inhalation [14]. This is acknowledged as a significant means for identifying health risks in human conducts and providing hazard proof for final decision people [15]. Due to significant differences in behavior and physiology, the study population was divided into children and adults [16].

#### 2.5.1. Dose–Response Assessment

The dose–response relationship refers to the relationship between the amount of PTEs to which an organism is exposed and the reaction that manifests in that organism [17]. This involves quantitative evaluation of the PTEs pollutants, and establishing their effects on the exposed population. The process allows for estimating the probability of the occurrence of adverse health reactions based on the quantitation of pollutant [18]. The difference in toxicity between non-carcinogens and carcinogens results in variations in the dose–effect relationship [19]. For non-carcinogenic substances, there is a minimum limit for the occurrence of health hazards. When the reference value is lower than this value, this indicates that it will not cause health hazards to the human body, and this limit is called the non-carcinogenic reference dose (RfD) [20]. For carcinogens, the carcinogenic strength factor (CSF) is used to express the level of carcinogenic probability. This paper adopted the “Technical Guidelines for Risk Assessment of Contaminated Sites” issued by China in 2014 (HJ 25.3-2014), the recommended values of pollutant parameters given in other documents, and the non-carcinogenic reaction dose and carcinogenicity of PTEs in different ways [21]. The strength coefficient is shown in Table 1 [22].

#### 2.5.2. Exposure Assessment

The daily exposure dose of the human body (CDI, mg·kg^−1^·d^−1^) was used to evaluate the exposure of PTEs in the arable land soil. The routes of human exposure to environmental pollutants include through the respiratory tract, skin, and mouth, specifically (1) inhalation and emissions of soil particles, (2) physical contact of the mouth with soil, and (3) incidental ingestion. The calculation formulas for each of the three routes are the definitions of parameters and specific values are shown in Table 2 [23].

(1) $$CDI_{ing}=\frac{C\times IR_{soil}\times EF\times ED}{BW\times AT\times 10^{6}}$$

(2) $$CDI_{Dermal}=\frac{C\times SA\times PE\times AF\times ABS\times ED}{BW\times AT\times 10^{6}}$$

(3) $$CDI_{inh}=\frac{PM_{10}\times M_{PM}\times ET\times IR_{air}\times EF\times ED}{PEF\times BW\times AT\times 10^{6}}$$

#### 2.5.3. Risk Characterization

The risk characterization is used to determine the non-carcinogenic risk and carcinogenic risk of PTEs pollutants to the human body and is calculated using the corresponding formula [24].

**1.**  **Non-carcinogenic risk**

The non-carcinogenic risk of human PTEs is expressed by the hazard quotient (HQ), which is the ratio of the daily human exposure dose (CDI, mg·kg^−1^·d^−1^) to the reference dose (RfD, mg·kg^−1^·d^−1^) [25]. The specific calculation formula is as follows

(4) $$HQ=\frac{CDI}{RfD}$$

(5) $$HI=\overset{n}{\underset{i=1}{\sum }}HQ_{i}=HQ_{Ing}+HQ_{Dermal}+HQ_{Inh}.$$

In the formula, HQ is the risk quotient; RfD is the contrast value dose of PTEs (mg/kg/day); and HI is the overall potential risk index. When HI > 1, non-carcinogenic risks may happen, and the eventuality increases with the increase in this value; otherwise, no non-carcinogenic effects are indicated [26].

**2.**  **Carcinogenic risk**

The carcinogenic risk is the recurring eventuality of cancer in a person’s lifetime, which can be calculated using the following formula

(6) CR=CDI×CSF

(7) $$TCR=\overset{n}{\underset{i=1}{\sum }}CDI_{i}\times CSF_{i}$$

In the formula, CR is the eventuality of carcinogenic risk, TCR is the total eventuality of carcinogenic risk, CSF is the carcinogenic intensity coefficient of each PTEs, and the total probability of carcinogenic risk is the sum of the three carcinogenic risk probabilities [27]. Combined with the 10^−6^ carcinogenic risk evaluation standard recommended by the “Technical Guidelines for Risk Assessment of Contaminated Sites” formulated by the Ministry of Environmental Protection and the USEPA standard, 10^−6^ to 10^−4^ are usually considered acceptable hazard intervals, namely, when CR < 10^−6^, it indicates that there is a negligible carcinogenic risk, or it can be said that there is no carcinogenic risk; when 10^−6^ < CR < 10^−4^, this indicates an acceptable carcinogenic risk, which is a virtual safety level, that is, it will not affect the exposed population. The level of risk that produces undesirable or harmful health effects is when CR > 10^−4^; the carcinogenic risk exceeds the acceptable range, and there may be a greater risk of carcinogenesis [28].

### 2.6. Pre-Determination of Suspected Pollution Sources

#### 2.6.1. Natural Factors

**Figure 2:**
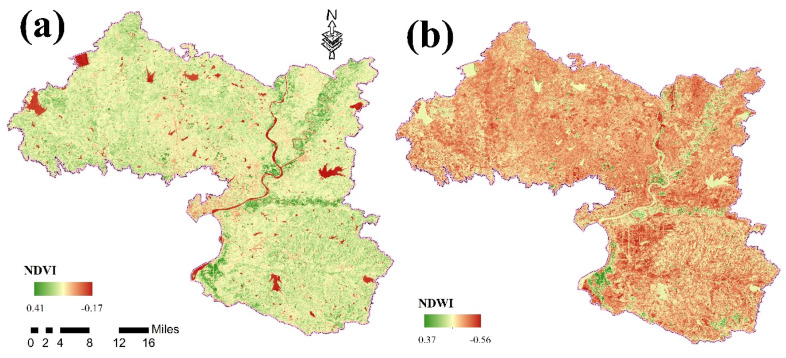

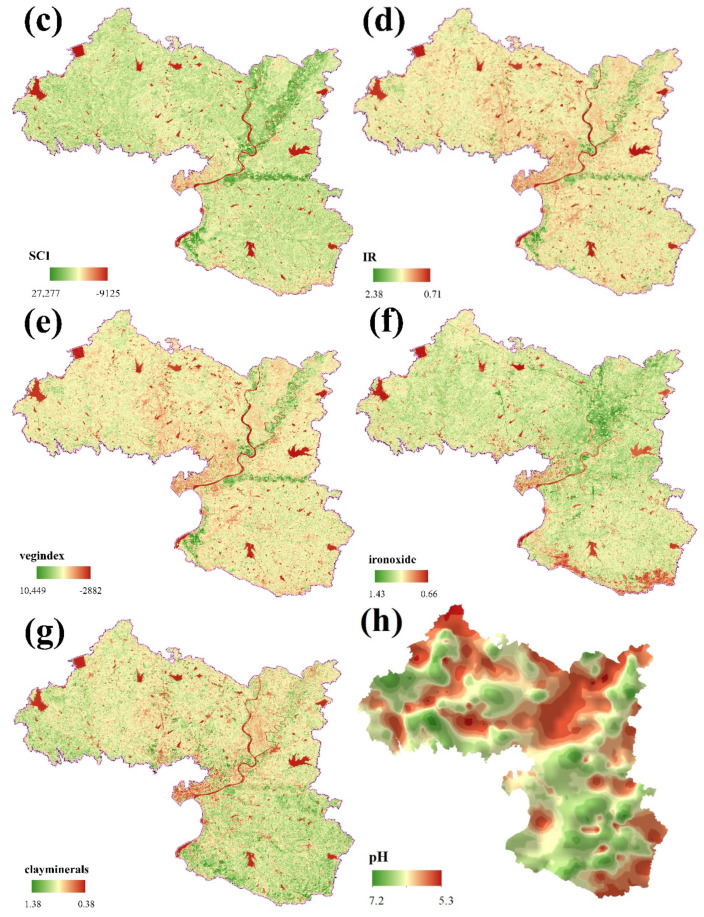

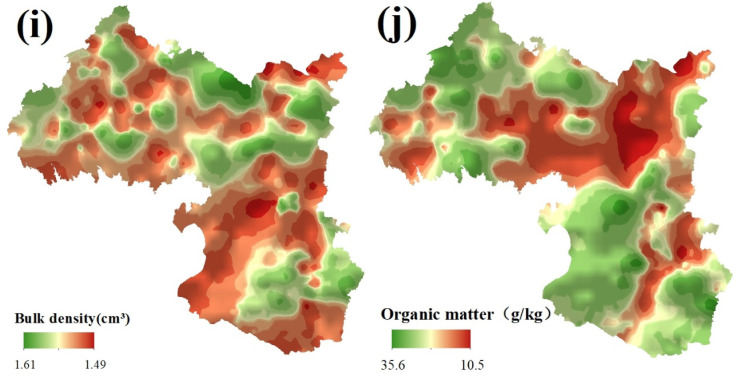
The spatial distribution of natural factors in the research region: (**a**) normalized difference vegetation index (NDVI); (**b**) normalized water index (NDWI); (**c**) soil color index (SCI); (**d**) ratio vegetation index (RVI); (**e**) Vegindex; (**f**) Ironoxide; (**g**) Clayminerals; (**h**) pH; (**i**) Bulk density; (**j**) Organic matter.

The Landsat 8 remote sensing image was processed and analyzed by ENVI software (Boulder, CO, USA), and the impact factors (normalized difference vegetation index (NDVI), normalized water index (NDWI), and soil color index (SCI)), Ratio vegetation index (RVI), vegindex, ironoxide, and clay mineral study area were obtained (Figure 2).

The use of NDVI is a standardized method for measuring healthy vegetation. Its value range is generally between −1 and 1. The greater the positive value, the greater the vegetation coverage. A value of 0 indicates that the surface is rocky or bare vegetation. Negative values indicate that the land surface is covered by water, such as from snow or rivers. The NDWI can highlight information about water in the image to obtain the moisture content of the vegetation canopy, which affects the content of PTEs in the soil.

The SCI characterizes the content of humus in the soil. Due to the ability of humus to adsorb metal ions [29], it will effectively reduce accumulation and harm resulting from PTEs in the soil [30]. Due to the vegetation coverage in the study area being generally higher, compared with other vegetation indexes, RVI is more sensitive to high-coverage vegetation areas and has the best correlation with biomass [31]. Vegindex is very sensitive to changes in the soil environment, which is conducive to monitoring the changes in the vegetation ecological environment, and can also better reflect the variation in the soil environment [32]. The index has a wide range of uses and reflects detailed information on the surface. Ironoxide is an important component of yellow and red soils [33]. Yellow soil contains more hydrated Ironoxide. Red soil forms iron bauxite under the action of desiliconization and aluminum enrichment. Therefore, Ironoxide is important when distinguishing soil types [34]. Clayminerals can determine the type of minerals in the area; the adsorption of clay has an important impact on the properties of the soil and the content of trace elements [35]. The pH value of the soil is one of the significant prerequisites that controls the utility of PTEs in the soil [36]. The soil bulk density is an important parameter of the soil structure. Its most important use is for calculating the soil porosity and soil permeability. After PTEs enter the soil, they take different chemical forms through dissolution, precipitation, complexation, and adsorption [37]. The complexation of soil organic matter with PTEs ions has an extremely important effect on the fixation and migration of PTEs ions in soil and water [38].

#### 2.6.2. Artificial Factors

**Figure 3:**
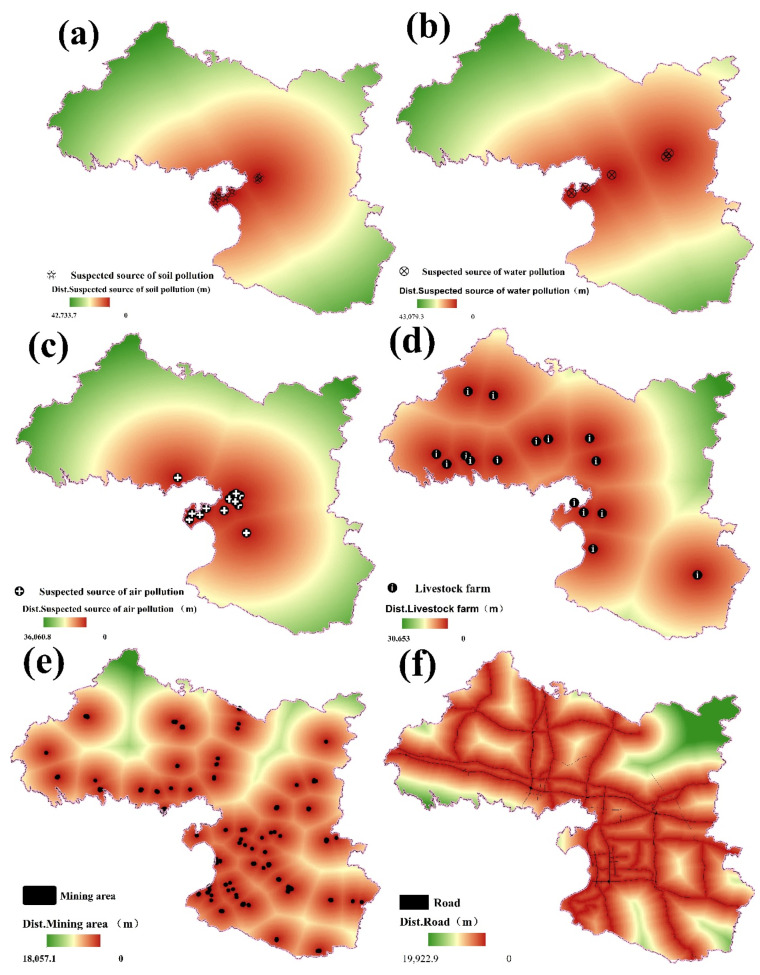

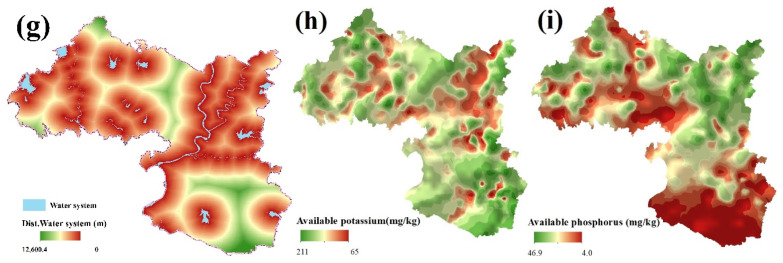
The spatial distribution of artificial factors in the research region: (**a**) suspected soil pollution sources; (**b**) suspected water pollution sources; (**c**) suspected air pollution sources; (**d**) livestock farm; (**e**) mining area; (**f**) road; (**g**) water system; (**h**) available potassium; (**i**) available phosphorus.

Via euclidean distance analysis of the mining areas; livestock farms; potential sources of air, water, and soil pollution; roads; and water systems in ArcGIS, a software of GIS, the distances between the 1109 sample points and nearby water systems, construction land, roads, factories, and enterprises were measured. Suspected soil pollution sources refer to the production threats to the surrounding environment by factories and enterprises in the form of transportation and storage, such as lead storage battery processing plants, auto part production plants and metal pipeline material processing plants; suspected sources of water pollution refer to factories and enterprises, sewage treatment, discharge, etc., that threaten the surrounding environment. These include sewage treatment companies and food processing plants, and suspected water pollution sources refer to factories and enterprises that threaten their surrounding environment in the form of exhaust gas emissions and atmospheric deposition, such as edible oil processing plants, electric vehicle production plants, etc. The distances were used in random forest regression analysis to quantitatively study the impact of sources, as shown in Figure 3.

## 3. Results

### 3.1. Spatial Distribution of PTEs in Soil

Ordinary kriging in the geostatistical method of ArcGIS software was used to draw and predict the spatial distribution of various PTEs in the soil and does not require the data to be normally distributed [39]. This is the best unbiased prediction method for considering the prediction of weighted averages, and this method can not only minimize the error variance of prediction but also reduce the workload of survey sampling [40]. When the study area is under obvious man-made influences, the distribution of abnormally high values of certain PTEs in the soil typically has a good correlation with the distribution of the industry, town, agriculture, and other activities linked with this type of pollution. Therefore, the spatial variation scale of PTEs can be used as an important basis for judging the source of each PTEs: the spatial variation scale caused by natural factors is relatively large, and the human contribution is mainly reflected in the scale of small and medium spaces [41].

**Figure 4:**
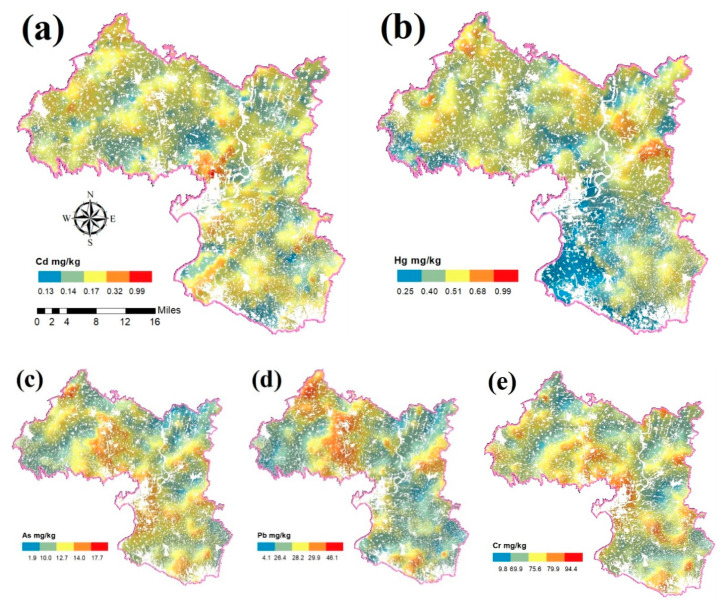
The spatial distribution of PTEs in the research region: (**a**) Cd; (**b**) Hg; (**c**) As; (**d**) Pb; and (**e**) Cr.

Figure 4 shows that the spatial distribution of the PTEs content in the study area’s soil has a patchy characteristic, and the distribution law is more distinct. The areas with soil containing high content of the five PTEs are mainly distributed near towns. The spatial distribution characteristics of the content of the PTEs Cd, As, and Cr are relatively similar, showing high values in the central region, where the central city is located. The spatial distribution of Hg content showed a trend of being high in the north and low in the southwest, and the Pb content spatial distribution indicated it was high in the northwest and south, and low in the middle.

### 3.2. Descriptive Statistical Analysis of Soil PTEs

The main statistics for PTEs in study areas are shown in Table 3. The soil pH in Xiangzhou is in the range of 4.7–7.3, indicating that the cultivated land in the study area is mostly in an acidic environment. The average value concentrations of all PTEs, beyond As and Pb, were underneath the Hubei Province’s background values [42]. Enrichment factors (EFs) were used to appraise the contamination levels and to assess the level of artificial influence [43]. The pollution level (EFs) is the quotient of the measured element value and its background value. The results show that As and Pb were accumulated to a certain degree. Therefore, we conclude that the agricultural land within research region is mildly polluted by artificial behavior.

### 3.3. Health Risk Assessment Results

Using the measured data for the content of PTEs in the soil of the research area, the potential hazard risks (HI) and total risk (TCR) of the five PTEs for people exposed through different routes were calculated according to Formulas (4)–(7). The calculation results are listed in Table 4.

Combined with the data in Table 4, we can see that the total probability of non-carcinogenic risk (HI) for adults is generally greater than the total probability of carcinogenic risk (HI) for children. Values beyond 1 show that the public may experience non-carcinogenic effects [44], For adults and children, As, Pb, and Cr may be non-carcinogenic. The order of hazard indices was: soil ingestion > dermal contact > air inhalation. The ratio of HQ by air inhalation was only about 0.01%. The order of dedication of the last three pathways was the same as in other people’s research [45]. Children had higher non-carcinogenic risks than adults through soil ingestion and dermal contact of PTEs, revealing that they are more susceptible to environmental pollutants. This might be due to the behavioral and physiological characteristics of children, including hand-to-mouth activities in soil and higher respiration rates per unit body weight [46].

The carcinogenic risks come from soil ingestion occupied for 99.95% of the TCR for adults and children, separately, which were higher than the carcinogenic risks owing to other pathways in general. For the total carcinogenic risk (TCR), the TCR of adults is generally greater than the TCR of children. The determined total carcinogenic risk of four of the PTEs in adults and children is, in descending order, Cr > As > Cd > Pb, where the carcinogenic risk caused by Cd, Cr, As is beyond the acceptable range, and Pb is at an acceptable carcinogenic risk at a virtual safety level, that is, the risk of not having adverse or harmful health effects on the exposed population level. The carcinogenic risks (Cd, As, Cr) for both adults and children were higher than the maximum acceptable risk (10^−4^) [47]. Children showed higher carcinogenic risks than adults. Measures should be taken to reduce carcinogenic risk.

### 3.4. Semivariogram Result

The parameters of the semivariogram model used to map and predict the content of various PTEs in the soil based on the ordinary kriging method are shown in Table 5. The percentage of the nugget value C_0_ to the base station value (C_0_+ C) is the nugget ratio (C_0_/(C_0_+ C)), which is generally used as a measure of the degree of the spatial correlation of variables [48]. If the nugget ratio is less than 0.25, the spatial correlation degree of the variable is strong, if the nugget ratio is between 0.25 and 0.75, the variable spatial correlation degree is medium, and if the ratio is greater than 0.75, the spatial correlation degree is weaker [49]. This indicates that, as the nugget ratio increases, the spatial correlation degree of the variables decreases, and the influence of artificial factors on the spatial variability of PTEs increases [50].

Among the five PTEs, the nugget value of the soil PTEs Cd and Hg was low, showing a strong spatial correlation, which indicates that the content of Cd and Hg in the soil was primarily influenced by the variation of composition, such as soil genre. External factors had the least influence. The nugget ratio of As, Pb and Cr content in the soil belonged to the medium-intensity variability, indicating that the spatial distribution of the As, Pb and Cr content in the soil in the research area was influenced by combined structural and random factors, and the spatial distribution shows clear human interference, which weakens the correlation of the spatial distribution of the soil As, Pb and Cr content, and the soil Cr content was the most affected.

### 3.5. Random Forest Simulation

A random forest regression model was set up with the Random Forest toolkit in R; the dependent variables are the content of the five PTEs at the sample points, and the independent variables are natural and artificial factors. After accurate and repeated tests, the most suitable effect was acquired when the calculation of decision trees was 1000, and the account of predictor variables selected by each point of intersection was 3. A higher weight of a factor shows its greater dedication to the cumulation of PTEs in the soil.

To further study the sources of PTEs, the RF toolkit in R (a software for statistical analysis, New York, NY, USA) was used to set up a random forest regression pattern. The 1109 samples points of the cultivated land in the research area were randomly separated into the train set and test set. The train set was used to build the random forest regression model, and the test data were used to confirm the accuracy of the model. In this study, the train set to test set ratio was 8:2, whereby 780 samples were selected for training, and 195 samples were selected for testing. The result of RF showed a proportion of illustrated variance higher than 70%, with correlation coefficients (R^2^) of 94% (Cd), 84% (Hg), 88% (As), 88% (Pb), and 90% (Cr), showing high prediction accuracy. Table 6 shows the dedication rate of the natural and artificial factors.

**Figure 5:**
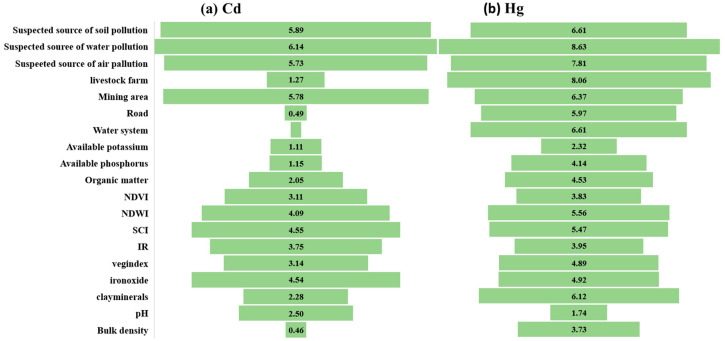

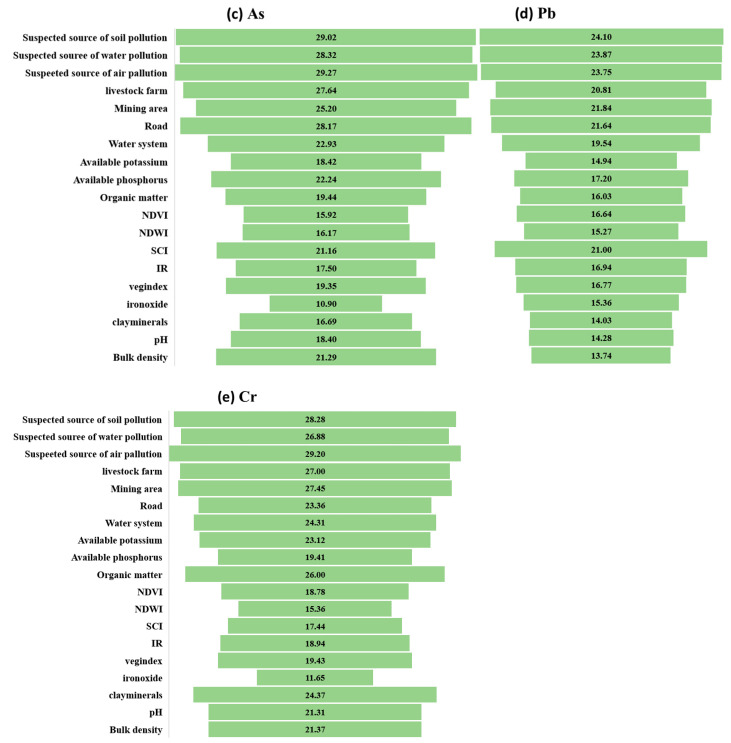
Plots of the variable importance measures (% increase in MSE) in Xiangzhou. (**a**) Cd; (**b**) Hg; (**c**) As; (**d**) Pb; and (**e**) Cr.

In Figure 5, the mean squared error (MSE) shows the weights of artificial influential conditions. The four most important factors explaining the Cd content were: distance from suspected source of water pollution (6.14%), distance from suspected source of soil pollution (5.89%), distance from mining area (5.78%), and distance from suspected source of water pollution (5.73%). The four most important factors explaining the Hg content were: distance from suspected source of water pollution (8.63%), distance from livestock farm (8.06%), distance from suspected source of air pollution (7.81%), and distance from suspected source of soil pollution (6.61%). The four most important factors explaining the As content were: distance from suspected source of air pollution (29.27%), distance from suspected source of soil pollution (29.02%), distance from suspected source of water pollution (28.32%), and distance from road (28.17%). The four most important factors explaining the Pb content were: distance from suspected source of air pollution (24.10%), distance from suspected source of soil pollution (23.87%), distance from suspected source of water pollution (23.75%), and distance from road (21.84%). The four most important factors explaining the Cr content were: distance from suspected source of air pollution (29.20%), distance from suspected source of soil pollution (28.28%), distance from suspected source of mining area (27.45%), and distance from a livestock farm (27.00%).

## 4. Discussion

### 4.1. Spatial Distribution Characteristics of PTEs Pollution

Characterizing the spatial distribution characteristics of PTEs pollution is an effective means to identify areas with high soil pollution and sources of pollution [51]. The spatial distribution characteristics of the content of the PTEs Cd, As, and Cr are similar, showing high values in the central region, which is the location of the central city; the spatial distribution of Hg content shows a trend of being high in the north and low in the southwest. The Pb content spatial distribution characteristics were high in the northwest and south, and low in the middle. This may be because most battery production plants and copper (tube and wire) production plants are mainly centralized in the middle and south of the study area, and only a small number of small copper (tube and wire) production plants are located in the north of the study area. Waste gas and wastewater is discharged during the production process [52]. The PTEs enter the soil, causing pollution of the soil [53]. In addition, the topography of this area is high in the northwest and south, and low in the middle [54]. PTEs accumulate in the middle due to the erosion of surface runoff, which further intensifies the distribution of PTEs in the research district in the middle [55].

### 4.2. Source Analysis of Pollution with PTEs

At present, large quantity studies have been conducted on the source of PTEs. Soil PTEs come from either natural or artificial activities. Naturally occurring PTEs are influenced by many factors, for instance, mineral ingredient, particle size, and organic carbon in soil [56]. Redistribution of PTEs in arable land soil can be influenced by surface runoff, weathering, erosion, etc. However, most studies have shown that pollution due to the PTEs mainly comes from human activities, as these are anthropogenically sourced metals [57], primarily resulting from industrial activities and sewage irrigation and the application of chemical fertilizers [58].

In this study, the random forest results proved that the main influencing factors of PTEs pollution were the already suspected air, soil, and water sources, which further verifies the spatial distribution results of the above five PTEs—that is, the closer the distance to the suspected source of pollution, the higher the content of PTEs. The industry and agriculture have developed rapidly in recent years; the research area is an industrial area that combines battery production, metal tube and wire production, and welding. Based on the existing literature, the enrichment of PTEs near the industrial areas are enormously affected by the deposition of metal containing dust [59]. In this study, the higher values of their spatial contents were in similar positions, all near the industrial production and processing areas, mining areas, etc. There are several battery production plants in the research area; in the production process of cadmium nickel and other batteries, the production of pole pieces requires a large amount of cadmium oxide and nickel. The production process of cadmium oxide will produce oxidation cadmium fumes, and the cadmium oxide needs to be cooled and washed with water [60]. The production of cadmium sponge, a battery filling material, also requires a large amount of water [61]. According to relevant data, every 1 ton of sponge cadmium produced will produce dozens of tons of cadmium-containing wastewater [62]. This cadmium-containing wastewater might drain directly to the fields or through the surface and ground water runoff, causing soil Cd pollution [63]. Xiangzhou is a significant carriage pivot in Hubei Province, although it has a high road network density and frequent vehicle traffic, and vehicle exhaust emissions will also have a certain impact on the soil environment near the road [64]. The protracted use of farm chemicals and manure and the discharge of domestic sewage are all cumulative elements influencing the levels of PTEs in agricultural land [65].

## 5. Conclusions

(1) For adults and children, the carcinogenic risk caused by Cr, As, and Cd was beyond the acceptable range that is expected to produce adverse or harmful health effects to the exposed population. As, Pb, and Cr in Xiangzhou’s cultivated soil may have non-carcinogenic risks (HI > 1), three soil elements posed potential noncarcinogenic risks to both adults and children via soil inhalation, dermal contact, and direct/indirect ingestion. Soil ingestion, the most significant exposure route, contributed about 99% of the health risks of PTEs. More heed should be paid to this sideways exposure route;

(2) Among the five PTEs, the nugget value of the soil PTEs Cd and Hg was low, showing a strong spatial correlation, which indicates that the content of Cd and Hg in the soil was mostly affected by natural structural factors such as the soil genre. The influence of variation was the least influential of the external factors. The nugget ratio of the As, Pb and Cr content in the soil was of medium intensity variability, indicating that the spatial distribution of the As, Pb and Cr content in the soil in the research area was influenced by structural and random factors. The spatial distribution showed clear human interference, which weakens the correlation of the spatial distribution of the soil As, Pb and Cr content, and the soil Cr content was the most affected;

(3) There were four main sources of these PTEs. Suspected water pollution sources were the main factors affecting Cd, Hg, As, and Pb levels, and suspected soil and air pollution sources were the common sources of the five PTEs. Agricultural activities, such as the excretion of livestock manure, were important determining factors for Hg. Industrial and mining factors were important in affecting the Cr content.

## Figures and Tables

**Table 1:** Non-carcinogenic response doses of six non-carcinogenic potentially toxic elements (PTEs) and the carcinogenic intensity coefficients of two carcinogenic PTEs.

	Element	Cd	Hg	As	Pb	Cr
RfD (mg·kg^−1^·d^−1^)	Respiratory intake	1.00 × 10^−5^	3.00 × 10^−4^	1.50 × 10^−5^	3.52 × 10^−3^	2.86 × 10^−5^
	Skin intake	2.50 × 10^−5^	3.00 × 10^−4^	1.23 × 10^−4^	5.25 × 10^−4^	6.00 × 10^−5^
	Oral intake	1.00 × 10^−3^	3.00 × 10^−4^	3.00 × 10^−4^	3.50 × 10^−3^	3.00 × 10^−3^
CSF (kg·d^−1^·mg^−1^)	Respiratory intake	1.8		4.3	0.042	84
	Skin intake	0.38		0.03		0.001
	Oral intake	6.1		1.5		0.5

**Table 2:** The meaning of each exposure assessment parameter and its specific value.

	Description	Value	
		Children (3–12 Years Old)	Adult (≥18 Years Old)
$PM_{10}$	Atmospheric particulate matter in adjacent areas in the research region, mg/m^3^	0.146	0.995
$M_{PM}$	The consistence of particulate matter in the air; because the particulate matter in the air comes from the soil, it is assumed to be equal to the concentration of PTEs in the soil		
ET	Exposure time, h/day	0.56	3.3
$IR_{air}$	Average daily air intake, m^3^/day	9.7	15.7
EF	Exposure frequency, day/year	350	
ED	Exposure duration, year	9	30
C	Concentration of PTEs in soil, mg/kg	Experimental measurement	
SA	Surface area of soil in contact with skin, cm^2^/day	8880	16,000
PE	Proportion of skin contact with soil	39.30%	
AF	Soil adhesion factor, mg/cm^2^	0.2	0.07
ABS	Skin absorption efficiency factor, dimensionless	Cd, Hg, Pb, Cr is 0.001; As is 0.03	
$10^{6}$	Conversion factor from kg to mg		
BW	Weight, kg	23.24	60.6
AT	Average contact time, day	Carcinogenic: 70 × 365; Non-carcinogenic: ED × 365	
$IR_{soil}$	Average daily intake of soil, mg/d	87	50

**Table 3:** Compendium statistics of concentrations of PTEs in soil at 1109 points in Xiangzhou.

	Cd	Hg	As	Pb	Cr
Maximum (mg/kg)	1.47	1.56	21.50	47.50	109.00
Minimum (mg/kg)	0.04	0.02	5.76	19.60	51.60
Mean (mg/kg)	0.14	0.05	12.89	29.23	78.58
Standard deviation, SD (mg/kg)	0.05	0.08	2.80	4.04	9.81
Coefficient of variation (%)	0.38	1.48	0.22	0.14	0.12
Background value	0.17	0.08	12.30	26.70	86.00
Enrichment factors	0.80	0.67	1.05	1.09	0.91

**Table 4:** The potential hazard risk (HI) and total risk (TCR) of five PTEs for people exposed through different routes.

		HQ			HI	CR			TCR
		Ing	Derm	Inh	Total	Ing	Derm	Inh	Total
Cd	Adult	1.20 × 10^−1^	1.21 × 10^−4^	1.34 × 10^−9^	1.20 × 10^−1^	3.14 × 10^−4^	4.92 × 10^−10^	1.03 × 10^−14^	3.14 × 10^−4^
	Child	5.45 × 10^−1^	4.99 × 10^−4^	3.65 × 10^−10^	5.45 × 10^−1^	4.27 × 10^−4^	1.55 × 10^−9^	8.45 × 10^−16^	4.27 × 10^−4^
Hg	Adult	1.56 × 10^−1^	3.93 × 10^−6^	1.74 × 10^−11^	1.56 × 10^−1^				
	Child	7.08 × 10^−1^	1.62 × 10^−5^	4.75 × 10^−12^	7.08 × 10^−1^				
As	Adult	37.70	6.94 × 10^−3^	1.26 × 10^−7^	37.70	7.27 × 10^−3^	1.10 × 10^−8^	2.32 × 10^−12^	7.27 × 10^−3^
	Child	171.00	2.87 × 10^−2^	3.44 × 10^−8^	171.00	9.90 × 10^−3^	3.46 × 10^−8^	1.90 × 10^−13^	9.90 × 10^−3^
Pb	Adult	7.33	1.23 × 10^−3^	8.10 × 10^−10^	7.33			5.14 × 10^−14^	5.14 × 10^−14^
	Child	33.20	5.08 × 10^−3^	2.22 × 10^−10^	33.30			4.21 × 10^−15^	4.21 × 10^−15^
Cr	Adult	23.00	2.89 × 10^−2^	2.68 × 10^−7^	23.00	1.48 × 10^−2^	7.43 × 10^−10^	2.76 × 10^−10^	1.48 × 10^−2^
	Child	104.00	1.20 × 10^−1^	7.33 × 10^−8^	104.00	2.01 × 10^−2^	2.35 × 10^−9^	2.26 × 10^−11^	2.01 × 10^−2^

**Table 5:** The fitting model and parameters of spatial variation semivariogram of PTEs.

PTEs	Model	Nugget Value/C_0_	Partial Base Station Value/C	Abutment Value/(C_0_ + C)	Nugget Ratio/[C_0_/(C_0_ + C)]	Range km	Coefficient
Cd	stable	0.0004	0.0014	0.0018	0.2049	0.88	2.00
Hg	stable	0.0636	0.3167	0.3804	0.1673	0.01	2.00
As	stable	2.1634	5.3851	7.5485	0.2866	12.81	0.62
Pb	stable	0.0041	0.0120	0.0161	0.2554	6.86	0.47
Cr	stable	40.3058	61.9820	102.2877	0.3940	12.77	0.65

**Table 6:** Results of the variable importance measures (% increase in mean squared error (MSE)) in Xiangzhou.

% Increase in MSE	Cd	Hg	As	Pb	Cr
Suspected source of soil pollution	5.89	6.61	29.02	24.10	29.20
Suspected source of water pollution	6.14	8.63	28.32	23.87	28.28
Suspected source of air pollution	5.73	7.81	29.27	23.75	27.45
livestock farm	1.27	8.06	27.64	21.84	27.00
Mining area	5.78	6.37	25.20	21.64	26.88
Road	0.49	5.97	28.17	21.00	26.00
Water system	0.23	6.61	22.93	20.81	24.37
Available potassium	1.11	2.32	18.42	19.54	24.31
Available phosphorus	1.15	4.14	22.24	17.20	23.36
Organic matter	2.05	4.53	19.44	16.94	23.12
NDVI	3.11	3.83	15.92	16.77	21.37
NDWI	4.09	5.56	16.17	16.64	21.31
SCI	4.55	5.47	21.16	16.03	19.43
IR	3.75	3.95	17.50	15.36	19.41
vegindex	3.14	4.89	19.35	15.27	18.94
Ironoxide	4.54	4.92	10.90	14.94	18.78
Clayminerals	2.28	6.12	16.69	14.28	17.44
pH	2.50	1.74	18.40	14.03	15.36
Bulk density	0.46	3.73	21.29	13.74	11.65
